# Complete *De Novo* Assembly of Monoclonal Antibody Sequences

**DOI:** 10.1038/srep31730

**Published:** 2016-08-26

**Authors:** Ngoc Hieu Tran, M. Ziaur Rahman, Lin He, Lei Xin, Baozhen Shan, Ming Li

**Affiliations:** 1 David R. Cheriton School of Computer Science, University of Waterloo, Waterloo, Ontario, Canada; 2 Bioinformatics Solutions Inc., Waterloo, Ontario, Canada

## Abstract

*De novo* protein sequencing is one of the key problems in mass spectrometry-based proteomics, especially for novel proteins such as monoclonal antibodies for which genome information is often limited or not available. However, due to limitations in peptides fragmentation and coverage, as well as ambiguities in spectra interpretation, complete *de novo* assembly of unknown protein sequences still remains challenging. To address this problem, we propose an integrated system, ALPS, which for the first time can automatically assemble full-length monoclonal antibody sequences. Our system integrates *de novo* sequencing peptides, their quality scores and error-correction information from databases into a weighted de Bruijn graph to assemble protein sequences. We evaluated ALPS performance on two antibody data sets, each including a heavy chain and a light chain. The results show that ALPS was able to assemble three complete monoclonal antibody sequences of length 216–441 AA, at 100% coverage, and 96.64–100% accuracy.

Monoclonal antibodies are playing highly successful roles in therapeutic strategies due to their mechanisms of variations^1^. However, it is such variations that also have defied us from an automated system to sequence them till now. Each monoclonal antibody (mAb) sequence is a novel protein that requires *de novo* sequencing with no resembling proteins (for the variable regions) in the databases.

Beginning from the low-throughput sequencing methods using Edman degradation^2^, significant progress has been made in the past decades. Especially, liquid chromatography coupled with tandem mass spectrometry (LC-MS/MS) has become a routine technology in peptide/protein identification. The high throughput sequencing requires computational approaches for the data analysis, including *de novo* sequencing directly from tandem mass spectra^3^,^4^,^5^ and database search methods that use existing protein sequence databases^6^,^7^,^8^,^9^,^10^,^11^,^12^. More specifically, various versions of shotgun protein sequencing (SPS) used CID/HCD/ETD^13^,^14^,^15^,^16^,^17^,^18^,^19^ fragmentation methods and other techniques to increase the coverage, and have achieved significant progress in attempt to fully sequence proteins, especially antibodies. Other methods have assumed the existence of similar proteins^20^, a known genome sequence^21^, or combined top-down and bottom up approaches^22^. In spite of these efforts, full-length *de novo* sequencing from tandem mass spectra of unknown proteins such as antibodies remains a challenging open problem^16^,^17^.

Two hundred and eighty years ago, Leonhard Euler wondered how he could cross the Pregel River traveling through each of the seven bridges of Konigsberg exactly once. Euler’s idea has been widely adopted in the concept of de Bruijn graph that plays the central role in the problem of sequence assembly^23^. The powerful performance of de Bruijn graph has been demonstrated in major genome and transcriptome assemblers such as Velvet^24^, Trinity^25^, and others. In the field of *de novo* protein sequencing, the idea of de Bruijn graph has been used for spectral alignment (A-Bruijn) in ref. 18, and recently has been extended to top-down mass spectra (T-Bruijn)^19^. However, incomplete peptide fragmentation, missing or low coverage, and ambiguities in spectra interpretation still pose challenges to existing tools to achieve full-length *de novo* assembly of protein sequences. The best result in existing literatures can only produce contigs as long as 200 AA at up to 99% accuracy^16^.

Our paper settles this open problem by introducing a comprehensive system, ALPS, which integrates *de novo* sequencing peptides, their intensity and positional confidence scores, and error-correction information from database and homology search into a weighted de Bruijn graph to assemble protein sequences. ALPS overcomes *de novo* peptides sequencing limitations and, for the first time, is able to automatically assemble full-length contigs of three mAb sequences of length 216–441 AA, at 100% coverage, and 96.64–100% accuracy. More details of the ALPS system and the performance evaluation on two antibody data sets are described in the following sections.

## Results

**Figure 1:**
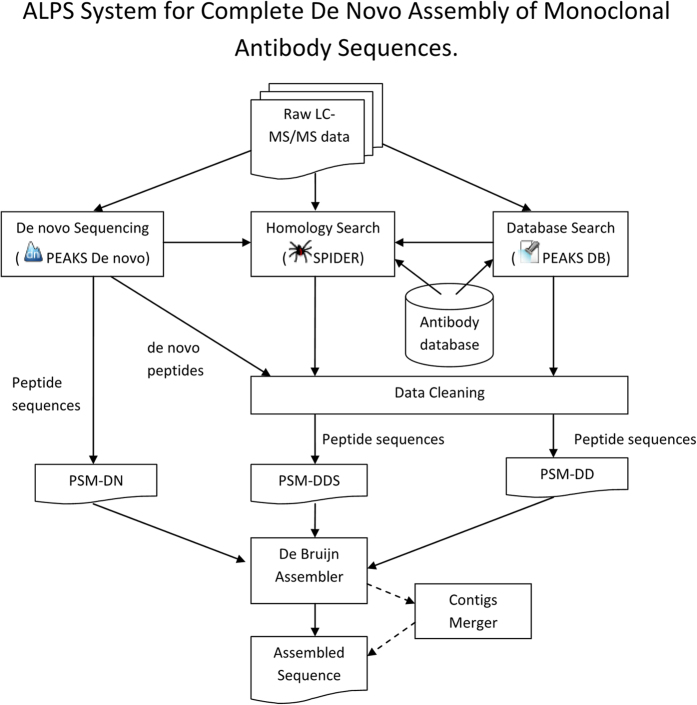
ALPS system for automated and complete *de novo* assembly of monoclonal antibody sequences.

Our ALPS system is outlined in Fig. 1. Briefly, antibody samples were first prepared according to the procedure described in Methods. Raw LC-MS/MS data were then imported into PEAKS Studio 7.5 for preprocessing (precursor mass correction, MS/MS de-isotoping and deconvolution, peptide feature detection). Subsequently, three following lists of peptides were generated for the assembly task. The first peptides list, PSM-DN, was generated from PEAKS *de novo* sequencing with precursor and fragment error tolerance as 10 ppm and 0.02 Da, respectively. Carbamidomethylation (Cys) was set as a fixed modification and oxidation (Met) and deamidation (Asn/Gln) as variable modifications. At most three variable modifications per peptide were allowed.

Next, PEAKS DB was used to identify peptide spectrum matches (PSMs) from existing protein databases. First, the data sets were searched against the UniProt database^26^ to identify the species and then a second search was performed against the in-house antibody database assembled for the identified species. Based on the current database search results and the *de novo* sequencing results from the first stage, a hybrid PSM set was generated as the second peptides list, PSM-DD, according to three criteria: 1) the scores of the PSMs identified by PEAKS DB must be higher than a specified threshold (in our case, which was selected with a false discovery rate (FDR) 1.0%); 2) the PSMs that were mapped to contaminant proteins must be filtered out; and 3) the Average Local Confidence (ALC) scores of PSMs identified from PEAKS *de novo* sequencing must be higher than 50 and the peptide sequence cannot be mapped a contaminant protein with more than seven amino acid residues. The motivation of using such a hybrid PSM set was to take advantage of database information to resolve amino acid assignment ambiguities of *de novo* sequencing peptides.

To take into account potential mutations in *de novo* sequencing peptides, the data sets were searched against the corresponding antibody database by using PEAKS SPIDER^27^. SPIDER tries to match *de novo* sequence tags with the database proteins and reconstructs a true sequence to minimize the sum of *de novo* errors and homology mutations between the true sequence and the one recorded in the database when a significant similarity is found. Finally, a hybrid PSM set containing PSMs from PEAKS *de novo* sequencing, PEAKS DB, and SPIDER were generated as our third peptides list, PSM-DDS. More details of the database search parameters can be found in Methods.

Three lists of peptides, PSM-DN, PSM-DD, and PSM-DDS were then imported into the de Bruijn graph assembler. In addition to the peptide sequences, we also incorporated peptides confidence scores and peptides intensities (feature areas) to form a weighted de Bruijn graph (Equation (1), Methods). Our experiments showed that those weight information played a crucial role to select the right paths for contigs extension and substantially improve the assembly quality.

Here we report our *de novo* assembly results for two datasets of monoclonal antibody sequences, each including a light chain and a heavy chain, hence a total of four samples. The first dataset, WIgG1, was generated from the LC-MS/MS analysis of the Intact mAb Mass Check Standard purchased from Waters. It is an intact mouse antibody purified by Protein-A with known molecular weights and amino acid sequences of both the light and heavy chains. The molecular weight and the target sequences can be readily used for the evaluation of our pipeline performance. The other dataset, HUMAN (IgG1) was generated from purified human antibody sample. This purified antibody sample has no amino acid sequences provided when purchased from SIGMA-Aldrich. To evaluate our pipeline, we manually worked out the amino acid sequences from the LC-MS/MS data with the assistance of PEAKS 7.5. The coverage and accuracy of both two target sequences were 100% guaranteed by the validation with three strict criteria: 1) The false discovery rate (FDR) at the peptide spectrum match (PSM) level was less than 0.1%; 2) Each amino acid was supported by at least 20 PSMs; 3) Each amino acid was supported by a pair of its fragmental ion peaks with at least 5% relative intensity.

**Figure 2:**
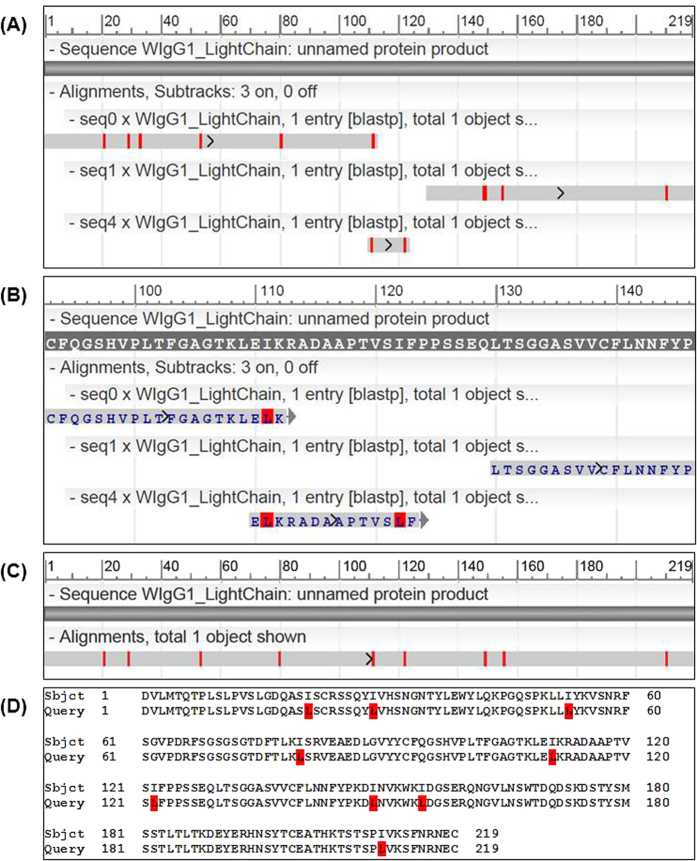
Assembly results for the WIgG1 light chain. (**A**) BLAST alignment of the top assembled contigs from list PSM-DN against the target light chain. (**B**) Zoom-in details of the alignment in (**A**). (**C**) BLAST alignment of the full-length contig assembled from list PSM-DD against the target light chain. (**D**) Details of the alignment in (**C**).

**Figure 3:**
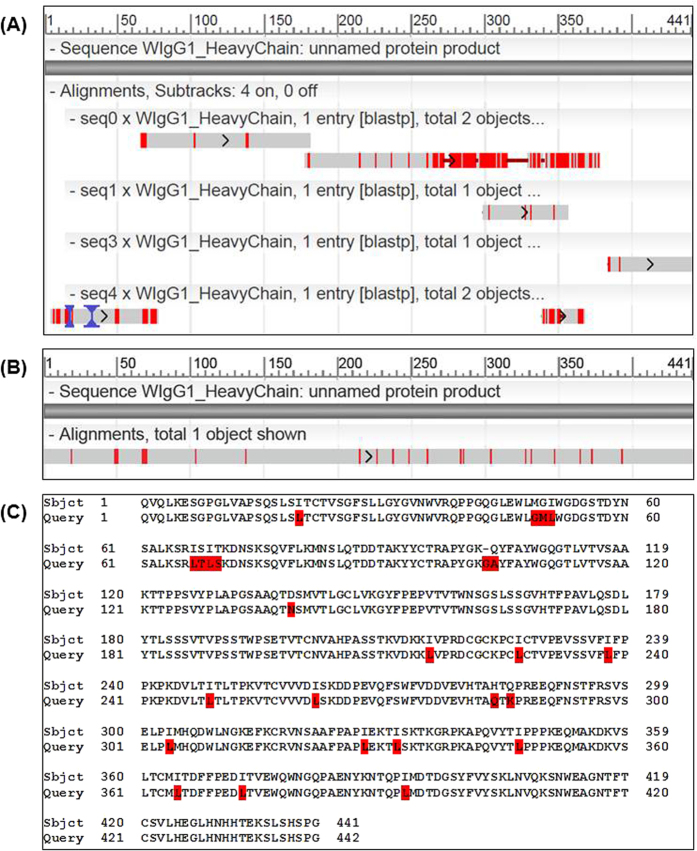
Assembly results for the WIgG1 heavy chain. (**A**) BLAST alignment of the top assembled contigs from list PSM-DN against the target heavy chain. (**B**) BLAST alignment of the full-length contig assembled from list PSM-DDS against the target heavy chain. (**C**) Details of the alignment in (**B**).

**Figure 4:**
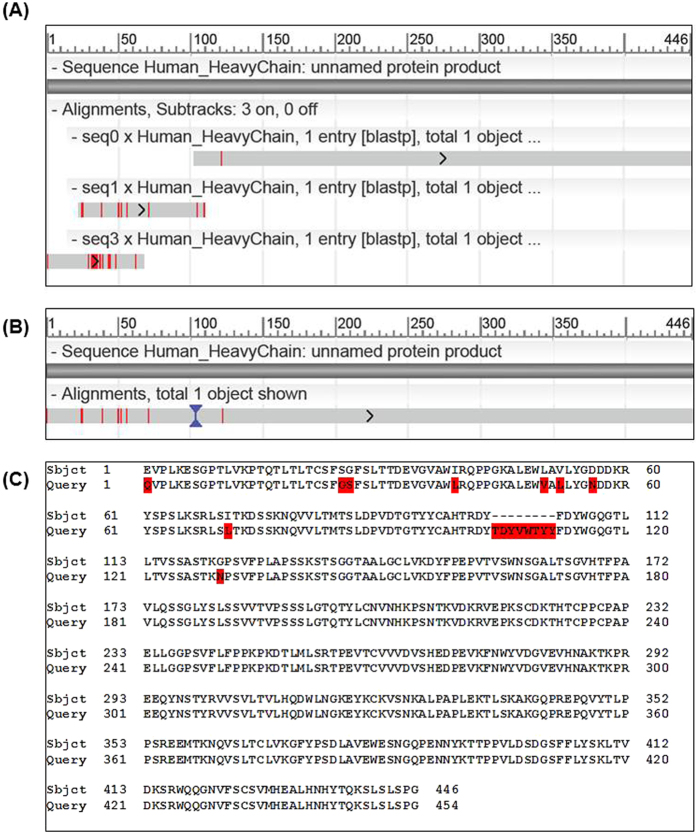
Assembly results for the HUMAN heavy chain. (**A**) BLAST alignment of the top assembled contigs from list PSM-DDS against the target heavy chain. (**B**) BLAST alignment of the template-alignment-based merging of PSM-DDS contigs against the target heavy chain. (**C**) Details of the alignment in (**B**).

The light chain lengths are in the range of 211–219 AA, while the heavy chains of IgGs are much longer, around 450 AA, and hence more challenging for the assembly task. For each sample, we prepared three lists of peptides PSM-DN, PSM-DD, and PSM-DDS, as described earlier, and then performed the assembly on each list. To evaluate the assembly results, we performed BLAST alignments of assembled contigs against the corresponding target sequences and then measured the coverage and accuracy. The assembly results are presented in Tables 1 and 2 and Figs 2, 3 and 4.

### Assembly Results for Dataset WIgG1

The light chain of this dataset has 219 AA. The de Bruijn assembly result from list PSM-DN with k = 6 is summarized in the BLAST alignment in Fig. 2A. We found that the first two contigs seq0 and seq1 retrieved from the de Bruijn assembler, with lengths 109 and 93 respectively, together covered 202 AA of the target light chain. The 17-AA gap between them is covered by some other lower-ranking contigs (for simplicity, only seq4 is shown in Fig. 2A). Our further investigation found that missing or low signal-to-noise of *de novo* peptides in that region prevented extension walks in the de Bruijn graph and thus produced such gaps. In particular, Fig. 2B shows the detailed alignment at that 17-AA gap. Seq0 could not be further extended to the right due to the missing of the 5-mer “LELKR” in list PSM-DN. On the other hand, seq1 was wrongly extended to the left after the 5-mer “LTSGG” due to an error of *de novo* spectra interpretation where “EQ” was wrongly interpreted as “DAA” in several *de novo* peptides.

Such limitations of *de novo* peptides can be handled by incorporating information from the database and homology search and using hybrid PSMs as described earlier. Indeed, from both lists PSM-DD and PSM-DDS, our de Bruijn assembler with k = 7 was able to retrieve the full-length contig of the WIgG1 light chain. Figure 2C shows the BLAST alignment of our assembled contig against the target sequence with 100% coverage and nine mismatches. The alignment details in Fig. 2D further show that all nine mismatches are I-to-L amino acids which have the same mass. Thus, our assembled contig also achieved 100% accuracy. For k = 6, the de Bruijn assembler made a wrong branching and hence caused a 2-AA deletion in the output contig (Supplementary Figure S1).

The WIgG1 heavy chain is 441-AA long, more than twice the light chain, and hence is more difficult for the assembly task. In Fig. 3A, we reported a few top contigs from the de Bruijn assembly result from list PSM-DN. The longest contig was 219-AA and covered two segments of total length 194 AA, that is, 43.99% of the target sequence. Other contigs also provided additional coverage. However, as can be seen from Fig. 3A, the problems of missing coverage and fragmentation were far more complicated for this heavy chain than for the light chain that we have seen earlier, and could not be solved by simply using *de novo* peptides alone. Hence, it is essential to include additional information from the database and homology search. Surprisingly, our de Bruijn assembler was able to assemble a 442-AA contig from list PSM-DDS that fully covered the target heavy chain (Figure 3B). To the best of our knowledge, this is the longest ever antibody sequence that has been automatically reconstructed in form of a single full-length contig from *de novo* peptides by using de Bruijn graph-based techniques.

The alignment details in Fig. 3C further show that our assembled contig from list PSM-DDS has one single insertion and twenty-four mismatches. Sixteen mismatches are I-to-L amino acids with the same mass. The consecutive insertion and mismatch at position 103 actually corresponds to one error of *de novo* spectra interpretation Q-to-GA. This would give our assembled contig an accuracy of 98.19% (433/441). However, we have strong evidence to believe that the target sequence provided by Waters has errors at positions 49–50, 68, 70 and our assembled contig indeed gives the correct amino acids at those positions. More explanations are available in Supplementary Figure S3. Hence, the accuracy of our assembled contig for the WIgG1 heavy chain is 99.09% (437/441).

The result from list PSM-DD, however, is slightly worse with a 12-AA insertion as the de Bruijn assembler made a wrong branching due to low signal-to-noise (Supplementary Figure S2).

### Assembly Results for Dataset HUMAN

For the HUMAN light chain of length 216 AA, our de Bruijn assembler again was able to obtain the full-length contig from the two lists PSM-DD and PSM-DDS at 100% accuracy. If only *de novo* peptides in list PSM-DN were used, the longest contig in the de Bruijn assembly result was 175-AA long, covering 170 AA (78.70%) of the target light chain (Supplementary Figure S4).

The HUMAN heavy chain is 446-AA long and was the most difficult among four sequences for our assembly task. Our best de Bruijn assembly result was obtained from list PSM-DDS and included three contigs of length 346, 92, 67, which together fully covered the target heavy chain (Fig. 4A). Even additional information from the database and homology search was not enough for our de Bruijn assembler to resolve the problems of missing coverage and low signal-to-noise of peptides and to produce a single full-length contig. Hence, we further combined the contigs retrieved from the de Bruijn graph by aligning them to a template protein sequence from the database that is most closely matched to those contigs. Based on that alignment and the positional confidence scores of the contigs, we merged them into the final sequence of length 454 AA. As shown in Fig. 4B,C, our assembled sequence has 9 mismatches two of which are I-to-L amino acids. The assembled sequence also has a 8-AA insertion at position 102. Hence, the accuracy of our assembled sequence is 96.64% (431/446) for this HUMAN heavy chain. Our further investigation found that the region of that 8-AA insertion is quite difficult to recover due to missing peptides coverage, even in the database and homology search.

### Assembly Results for Dataset of Chicken Lysozyme and Dataset of 6-protein Mixture

In addition to antibodies, we also evaluated the performance of ALPS on other types of proteins. It should be noted that the following proteins are known proteins and their sequences already exist in many databases, e.g. UniProt. Since our tool uses information from database and homology search, to avoid bias in the evaluation, we removed all those protein sequences and their homologs from the UniProt database before the experiment. The homolog protein groups were identified in PEAKS based on the Parsimony Principle.

We first tested ALPS on a small dataset of Chicken Lysozyme^20^. The protein sequence is 147-AA long, of which the first 18 AA were cleaved-off in processing, leaving the target sequence of length 129 AA^20^. Our tool was able to assemble a contig of length 130 AA that fully covered the target sequence at 100% accuracy.

Next, we tested ALPS on a more complicated dataset, a mixture of 6 proteins: murine leptin, human kallikrein-related peptidase, *E. coli* GroEL, horse heart myoglobin, bovine aprotinin, and horseradish peroxidase. More details of MS/MS experiments can be found in the original study^16^. Our assembly results from list PSM-DDS with k = 7 are summarized in Table 3 and Supplementary Figure S5. The lengths of 6 protein sequences are from 100–548 AA. The lengths of the longest contig assembled for each target protein sequence are from 65–444 AA. The sequencing coverage is from 65–99.4% and the sequencing accuracy is from 95.3–99.8%. In Table 3 we also compared our assembly results with those reported by Meta-SPS in the original study^16^. In general, our tool was able to assemble longer contigs (for 5/6 proteins), while the sequencing coverage and accuracy are comparable. This demonstrates the advantage of our integrated system to generate long contigs for the *de novo* assembly of protein sequences.

## Discussion

*De novo* assembly of novel protein sequences is one of the most challenging problems in mass spectrometry-based proteomics. The main difficulties of this assembly task include limitations in peptides fragmentation and coverage, as well as ambiguities in spectra interpretation. To solve this problem, we proposed the integrated system ALPS to combine *de novo* sequencing peptides, their intensities and positional confidence scores, and error-correction information from database and homology search into a weighted de Bruijn graph to assemble protein sequences. We further demonstrated that such an integrated system indeed was able to overcome the aforementioned limitations and achieve the complete assembly goal.

We evaluated ALPS on two antibody data sets, each including a light chain and a heavy chain. As summarized in Table 1, we obtained three full-length contigs from the de Bruijn assembler for three of the four antibody sequences. The assembled contigs for the two light chains (with lengths 219 and 216 AA, respectively) achieved 100% accuracy, while the contig for the WIgG1 heavy chain (length 441 AA) achieved 99.09% accuracy. For the remaining HUMAN heavy chain (length 446 AA), we obtained three contigs of lengths 346, 92, 67, which together fully covered that heavy chain. The final sequence combined from those three contigs achieved 96.64% accuracy. In addition, we summarized in Table 2 that the *de novo* assembly results were substantially improved by integrating the information from database and homology search together with *de novo* peptides and their positional confidence scores. Finally, we tested ALPS on a dataset of chicken lysozyme and a dataset of 6-protein mixture and showed that ALPS was also applicable to a variety of proteins other than antibodies.

The de Bruijn graph is a state-of-the-art approach for sequence assembly and it has been widely used in high-throughput sequencing. The application of de Bruijn graph in proteomics, however, has been focusing on spectra alignment to address the challenge of spectra interpretation. Keeping tracks of spectra, however, has limited the ability of de Bruijn graph to extend the contigs. In our approach, we apply de Bruijn graph directly to peptide sequences rather than the spectra. All spectra information, together with additional information from database and homology search, are processed earlier at the *de novo* peptide sequencing step. Then, the input to de Bruijn graph includes peptides sequences and their positional confidence scores. This is the same concept as the case of high-throughput sequencing DNA/RNA reads and their base quality (phred) scores. As a result, our tool ALPS is able to assemble longer contigs than previous works, and can achieve full-length contigs that span the entire target sequences.

In conclusion, our ALPS system has solved the problem of automated and complete *de novo* assembly of monoclonal antibody sequences. Furthermore, we believe that ALPS can be further generalized for the *de novo* assembly of any novel proteins with appropriate databases and experiments setting. The performance of ALPS can be affected by the lack of overlapping peptides and the errors in *de novo* peptide sequencing. However, given the rapid advance of mass-spectrometry technologies, the peptide sequencing coverage and accuracy will be continuously improved, making ALPS a great resource for the *de novo* assembly of protein sequences.

## Methods

### Sample Preparation

#### SDS-PAGE

The heavy and light chains of an antibody were separated by SDS polyacrylamide gel electrophoresis (SDS-PAGE). Briefly, 0.5 μg of the antibody was placed, reduced, and denatured in gel loading buffer. The sample was subsequently loaded into three wells that contained a 10% precast gel (BioRad). The gel was subjected to 180 constant volts for 50 minutes. Following this, the gel was stained with Coomassie Blue. Gel bands that contained the antibody were excised.

### Deglycosylation and Endoprotease Digestion

Each excised band was reduced with dithiothreitol (DTT). Free cysteine residues were then alkylated using iodoacetamide. The heavy chain bands were deglycosylated with PNGase F (Roche Diagnostics) overnight using the manufacturer’s protocol. The pH was adjusted for each protease and three enzyme digestions were carried out overnight according to the manufacturer’s (Roche) instructions: 1) Asp N, 2) Chymotrypsin, 3) Trypsin. The peptides were extracted from the gel bands, desalted using C18 Zip-Tips^®^ (Millipore) and dried in a speed-vac.

### LC-MS/MS Analysis

The desalted peptides were suspended in 0.1% formic acid and 1/10 of each of the digests were subjected to LC-MS/MS analysis on a Thermo-Fisher Scientific Q-Exactive (Q-E) Orbitrap mass spectrometer. The gradient was supplied using a Thermo-Fisher EASY nLC-1000 UHPLC system and consisted of 0 to 40% acetonitrile in 0.1% formic acid over 1 hour at 250 nL per minute. The Q-E was run in a data dependent mode with 10 MS/MS events per cycle. The parent ion resolution was 70,000 FWHM and the fragment ion resolution was 17,500 FWHM. The 12 resulting raw data files (6 for each antibody, 3 for the light chain and 3 for the heavy chain) were used for data analysis.

### MS/MS Data Analysis

Our ALPS system is outlined in Fig. 1. More details of each analysis stage are described in the following sections.

### Data Processing and Cleaning

The raw data were first imported into PEAKS Studio 7.5, preprocessed (precursor mass correction, MS/MS de-isotoping and deconvolution, peptide feature detection), and analyzed to generate three following lists of peptides for the subsequent assembling.

#### PSM-DN: Results from De Novo Sequencing

The first stage was *de novo* sequencing from tandem mass spectra. PEAKS *de novo* sequencing was performed with precursor and fragment error tolerance as 10 ppm and 0.02 Da, respectively. Carbamidomethylation (Cys) was set as a fixed modification and oxidation (Met) and deamidation (Asn/Gln) as variable modifications. At most three variable modifications per peptide were allowed. The peptide sequences identified by the *de novo* sequencing analysis were exported along with their feature areas and positional confidence scores.

#### PSM-DD: Results from De Novo Sequencing and PEAKS DB

PEAKS DB^6^, the database search module in PEAKS Studio 7.5, was then used in the second stage to identify peptide spectrum matches (PSMs) from existing protein databases. To determine a confidence threshold for PSMs, the PEAKS-embedded target-decoy approach, “decoy fusion”^6^, was used to estimate the false discovery rate (FDR) of the PEAKS DB result. In our experiments, we assumed the species of the samples were unknown. Therefore, the data sets were searched first against the UniProt database^26^ to identify the species. Once the species was confirmed, a second database search was performed on the data sets against the in-house antibody database assembled for the identified species. Note that the antibody database used in the PEAKS DB search also includes 329 commonly observed contaminant proteins. This contaminant database contains proteins from the cRAP contaminant database^28^, the MaxQuant contaminant database^29^, and a few contaminants used in ABRF iPRG 2012 study. More specifically, the WIgG1 data sets were searched against the mouse antibody database and the HUMAN IgG1 data sets were searched against the human antibody database in our experiments. Other search parameters were kept the same as used in the respective *de novo* sequencing analysis. Based on the current database search results and the *de novo* sequencing results from the previous stage, a hybrid PSM set was generated for the subsequent antibody sequencing assembling according to three criteria: (1) the scores of the PSMs identified by PEAKS DB must be higher than a specified threshold (in our case, which was selected with FDR 1.0%); (2) the PSMs that were mapped to contaminant proteins must be filtered out; and (3) the Average Local Confidence (ALC) scores of PSMs identified from PEAKS *de novo* sequencing must be higher than 50 and the peptide sequence cannot be mapped a contaminant protein with more than seven amino acid residues. Each PSM in the hybrid set was also accompanied by its feature area and positional confidence scores for the subsequent assembling. The motivation of using such a hybrid PSM set was to take advantage of database information to resolve amino acid assignment ambiguities of *de novo* sequencing peptides.

#### PSM-DDS: Results from De Novo Sequencing and PEAKS DB and SPIDER

Biological samples for antibody sequencing commonly contain proteins with slightly different sequences to the ones recorded in the existing protein databases. Ignoring those mutated peptides can potentially lead to errors in the complete and accurate antibody sequencing. To detect amino acid variants, the data sets were searched by the SPIDER^27^, integrated in PEAKS software, against the given antibody database. SPIDER tries to match the *de novo* sequence tags with the database proteins and reconstructs a true sequence to minimize the sum of *de novo* errors and homology mutations between the true sequence and the one recorded in the database when a significant similarity is found. The PSMs reported by SPIDER are then filtered at 1.0% FDR. Similarly to the aforementioned hybrid PSM set in the previous stage, a PSM set containing PSMs from PEAKS *de novo* sequencing, PEAKS DB, and SPIDER were exported for the subsequent assembling.

### Weighted de Bruijn Graph Construction and Contigs Assembly

The three lists of peptides together with their intensities (feature areas) and positional confidence scores were obtained from PEAKS as described in the previous procedures. Subsequently, all possible k-mers were extracted from the peptides. Each k-mer was further split into two overlapping substrings of length k-1, called left and right (k-1)-mers. The left and right (k-1)-mers represent nodes in the de Bruijn graph while the k-mer corresponds to a directed edge in the graph, pointing from the left to the right (k-1)-mer. Our experimental results suggest that k = 6 or k = 7 are optimal for the assembly of antibody sequences. Using shorter k-mers will encounter the issue of repetitiveness in target sequences, while using longer k-mers will not have enough peptides coverage for the assembly task.

We found that the peptides’ intensities and positional confidence scores provide more useful information and substantially improve the assembly quality from the de Bruijn graph. In particular, we define the confidence score of each (k-1)-mer as the weighted geometric mean of its amino acids’ confidence scores. The weight of each (k-1)-mer was then calculated as the product of its confidence score and the intensity of the peptide from which the (k-1)-mer was extracted. Since a (k-1)-mer can appear in multiple peptides, its weight was accumulated over the processing of all those peptides. Our formulation of the node weights is defined in the following equation:


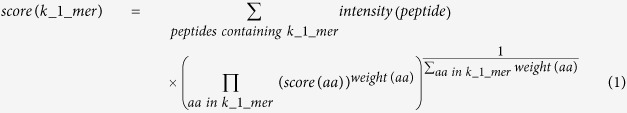


After the de Bruijn graph was constructed, contigs were assembled by performing greedy walks through the graph as following.

- Step 1: select the (k-1)-mer with the highest weight as the seed for the new contig.
- Step 2: extend the seed in both forward and backward directions by selecting the neighbors with the highest weights and concatenating the new amino acids to the current contig. This step was repeated until no further extension was possible. After a (k-1)-mer had been used for seed or extension, it was discarded from graph.
- Step 3: repeat steps 1 and 2 until the graph became empty or a desired number of contigs had been generated.

The assembly output was a list of contigs in the order that they were extracted from the graph. In addition, each contig was accompanied by positional confidence scores for its residues.

### Final Protein Sequence

If the de Bruijn assembler produced a few contigs rather than a single full-length one to cover the target sequence, the contigs were combined into the final sequence by using a template alignment. A template sequence that is most closely matched to the contigs was obtained from the database. Subsequently, the contigs were aligned to the template sequence to determine their relative positions to each other. Finally, the contigs were merged to one single sequence and their overlapping regions were resolved by using the corresponding positional confidence scores.

### Data Availability

The RAW files of two antibody datasets can be downloaded from the database MassIVE with accession number MSV000079801. The datasets of chicken lysozyme and 6-protein mixture can be obtained from the respective papers. All target sequences, antibody database, assembler tool, and assembly results are included in Supplementary Materials. More details are available in Supplementary Information.

## Additional Information

**How to cite this article**: Tran, N. H. *et al*. Complete *De Novo* Assembly of Monoclonal Antibody Sequences. *Sci. Rep.* **6**, 31730; doi: 10.1038/srep31730 (2016).

## Supplementary Material

Supplementary Information

Assembly results

## Figures and Tables

**Table 1:** Summary of ALPS *De Novo* Assembly Results on Antibody Datasets.

	WIgG1–Light (219 AA)	WIgG1–Heavy (441 AA)	Human–Light (216 AA)	Human–Heavy (446 AA)
Assembly Results	Full-length contig from de Bruijn assembler	Full-length contig from de Bruijn assembler	Full-length contig from de Bruijn assembler	3 contigs (lengths 346, 92, 67) from de Bruijn assembler; Complete sequence merged from 3 contigs
Target Sequence Coverage (%)	100.00	100.00	100.00	100.00
Target Sequence Accuracy (%)	100.00	99.09	100.00	96.64

The target sequence coverage was calculated as the percentage of amino acids of the target sequence that were covered by at least one contig. The target sequence accuracy was calculated as the percentage of matched amino acids. I-to-L were not considered as mismatched.

**Table 2:** Length (AA), Number of Amino Acids Recovered (AA), Target Sequence Coverage (%), and Contig Assembly Accuracy (%) of the Longest Contigs for Antibody Datasets.

	WIgG1–Light (219 AA)	WIgG1–Heavy (441 AA)	Human–Light (216 AA)	Human–Heavy (446 AA)
PSM-DN with frequencies	114; 109; 49.77; 95.61	143; 129; 29.25; 90.21	175; 170; 78.70; 97.14	98; 74; 16.59; 75.51
PSM-DN with weights	109; 109; 49.77; 100.00	219; 194; 43.99; 88.58	175; 170; 78.70; 97.14	154; 121; 27.13; 78.57
PSM-DD	219; 219; 100.00; 100.00	453; 441; 100.00; 97.35	216; 216; 100.00; 100.00	346; 344; 77.13; 99.42
PSM-DDS	219; 219; 100.00; 100.00	442; 441; 100.00; 99.77	216; 216; 100.00; 100.00	346; 344; 77.13; 99.42

The target sequence coverage was calculated as the percentage of amino acids of the target sequence that were covered by the respective longest contig. The contig assembly accuracy was calculated as the percentage of correct amino acids of the longest contig that were aligned to the respective target sequence.

**Table 3:** Summary of *De Novo* Assembly Results on 6-protein Mixture Dataset.

	leptin (167 AA)	kallikrein (261 AA)	groEL (548 AA)	myoglobin (154 AA)	aprotinin (100 AA)	peroxidase (353 AA)
Meta-SPS (with κ ≥ 1)						
Longest Contig (AA)	93	134	194	80	59	58
Sequencing Coverage (%)	86.2	87.7	92.5	92.2	64.0	67.4
Sequencing Accuracy (%)	100.0	98.5	97.7	99.3	80.0	100.0
ALPS (with list PSM-DDS, 7-mers)						
Longest Contig (AA)	131	77	444	118	65	92
Sequencing Coverage (%)	87.4	83.5	99.1	99.4	65.0	66.6
Sequencing Accuracy (%)	98.6	96.8	99.8	98.0	95.4	95.3

Sequencing coverage was calculated as the percentage of amino acids of the protein sequence that were covered by at least one contig. Sequencing accuracy was calculated as the percentage of all annotated sequence calls that were labeled correct. The Meta-SPS results were reported in ref. 16.
