# Supplementary material for: Complete De Novo Assembly of Monoclonal Antibody Sequences
Source: Sci Rep. 2016 Aug 26;6:31730. doi: 10.1038/srep31730 (PMC4999880; doi:10.1038/srep31730)
Supplement: Supplementary Information [file srep31730-s1.pdf]

Complete De Novo Assembly of Monoclonal Antibody Sequences

Ngoc Hieu Tran, M. Ziaur Rahman, Lin He, Lei Xin, Baozhen Shan, Ming Li

Supplementary Data

The supplementary data was submitted in a folder named “ALPS_Data” together with the manuscript. The folder “ALPS_Data” includes four subfolders for the four samples in our study. Each subfolder contains three csv files “peptides_0.csv”, “peptides_db.csv”, and “peptides_spider.csv”, corresponding to three peptides lists PSM-DN, PSM-DD, and PSM-DDS, respectively, in the manuscript. In addition, the subfolders also include the assembled contigs from three peptides lists and their positional confidence scores. The target light chains and heavy chains are also provided for evaluation. Finally, a java executable file is included in the folder “ALPS_Data” with detailed instructions how to perform the assembly on the peptides lists. The database that we used for the database and homology search is also provided.

Supplementary Figures

(A)

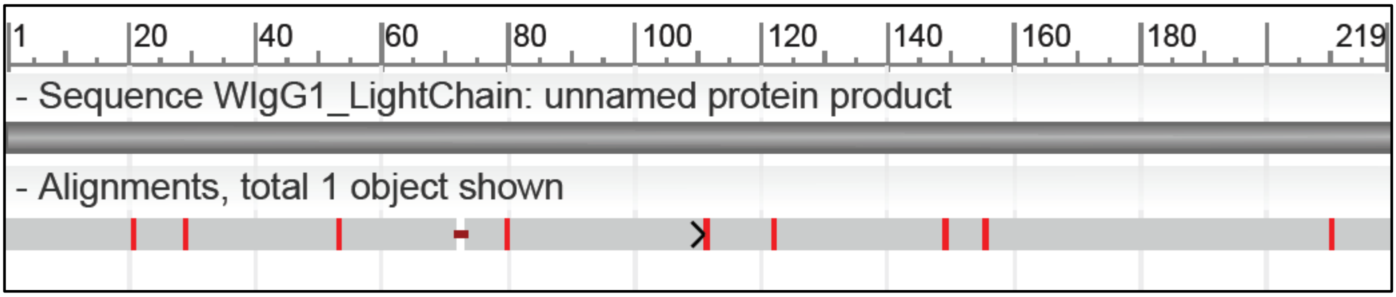

(B)

```
Sbjct  1    DVLMTQTPLSLPVSLGDQASISCRSSQYIVHSNGNTYLEWYLQKPGQSPKLLIYKVSNRF  60
Query  1    DVLMTQTPLSLPVSLGDQASLSCRSSQYLVHSNGNTYLEWYLQKPGQSPKLLLYKVSNRF  60

Sbjct  61   SGVPDRFSGSGSGTDFTLKISRVEAEDLGVYYCFQGSHVPLTFGAGTKLEIKRADAAPTV  120
Query  61   SGVPDRFSGSG--TDFTLKLSRVEAEDLGVYYCFQGSHVPLTFGAGTKLELKRADAAPTV  118

Sbjct  121  SIFPPSSEQLTSGGASVVCFLNNFYPKDINVKWKIDGSERQNGVLNSWTDQDSKDSTYSM  180
Query  119  SLFPPSSEQLTSGGASVVCFLNNFYPKDLNVKWKLDGSERQNGVLNSWTDQDSKDSTYSM  178

Sbjct  181  SSTLTLTKDEYERHNSYTCEATHKTSTSPIVKSFNRNEC  219
Query  179  SSTLTLTKDEYERHNSYTCEATHKTSTSPLVKSFNRNEC  217
```

Supplementary Figure S1. Assembly results from list PSM-DD with k=6 for the WIgG1 light chain. (A) BLAST alignment of the contig against the target light chain. (B) Details of the alignment in (A).

(A)

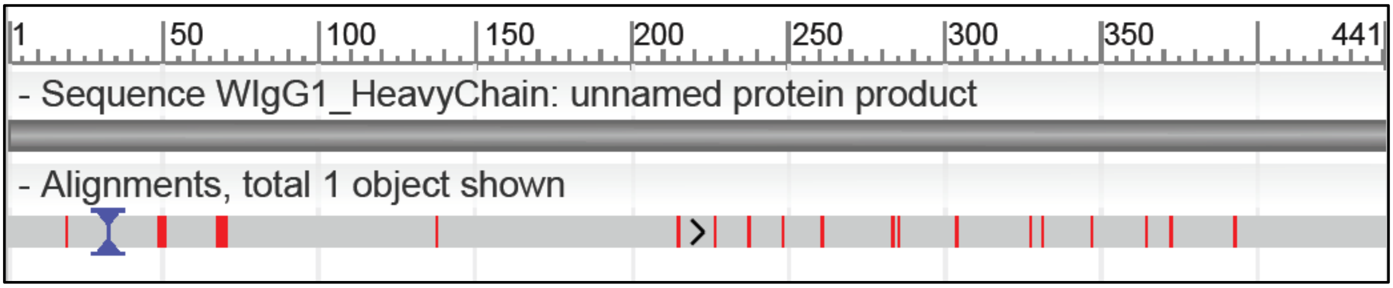

(B)

```
Sbjct  1    QVQLKESGPGLVAPSQSLSITCTVSGFSLLGY------------GVNWVRQPPGQGLEWL  48
Query  1    QVQLKESGPGLVAPSQSLSLTCTVSGFSLLGYNHHQMMCLSLGYGVNWVRQPPGQGLEWL  60

Sbjct  49   MGIWGDGSTDYNSALKSRISITKDNSKSQVFLKMNSLQTDDTAKYYCTRAPYGKQYFAYW  108
Query  61   GMLWGDGSTDYNSALKSRLTLSKDNSKSQVFLKMNSLQTDDTAKYYCTRAPYGKQYFAYW  120

Sbjct  109  GQGTLVTVSAAKTTPPSVYPLAPGSAAQTDSMVTLGCLVKGYFPEPVTVTWNSGSLSSGV  168
Query  121  GQGTLVTVSAAKTTPPSVYPLAPGSAAQTNSMVTLGCLVKGYFPEPVTVTWNSGSLSSGV  180

Sbjct  169  HTFPAVLQSDLYTLSSSVTVPSSTWPSETVTCNVAHPASSTKVDKKIVPRDCGCKPCICT  228
Query  181  HTFPAVLQSDLYTLSSSVTVPSSTWPSETVTCNVAHPASSTKVDKKLVPRDCGCKPCLCT  240

Sbjct  229  VPEVSSVFIFPPKPKDVLTITLTPKVTCVVVDISKDDPEVQFSWFVDDVEVHTAHTQPRE  288
Query  241  VPEVSSVFLFPPKPKDVLTLTLTPKVTCVVVDLSKDDPEVQFSWFVDDVEVHTAQTKPRE  300

Sbjct  289  EQFNSTFRSVSELPIMHQDWLNGKEFKCRVNSAAFPAPIEKTISKTKGRPKAPQVYTIPP  348
Query  301  EQFNSTFRSVSELPLMHQDWLNGKEFKCRVNSAAFPAPLEKTLSKTKGRPKAPQVYTLPP  360

Sbjct  349  PKEQMAKDKVSLTCMITDFFPEDITVEWQWNGQPAENYKNTQPIMDTDGSYFVYSKLNVQ  408
Query  361  PKEQMAKDKVSLTCMLTDFFPEDLTVEWQWNGQPAENYKNTQPLMDTDGSYFVYSKLNVQ  420

Sbjct  409  KSNWEAGNTFTCSVLHEGLHNHHTEKSLSHSPG  441
Query  421  KSNWEAGNTFTCSVLHEGLHNHHTEKSLSHSPG  453
```

Supplementary Figure S2. Assembly results from list PSM-DD for the WIgG1 heavy chain. (A) BLAST alignment of the contig against the target heavy chain. (B) Details of the alignment in (A).

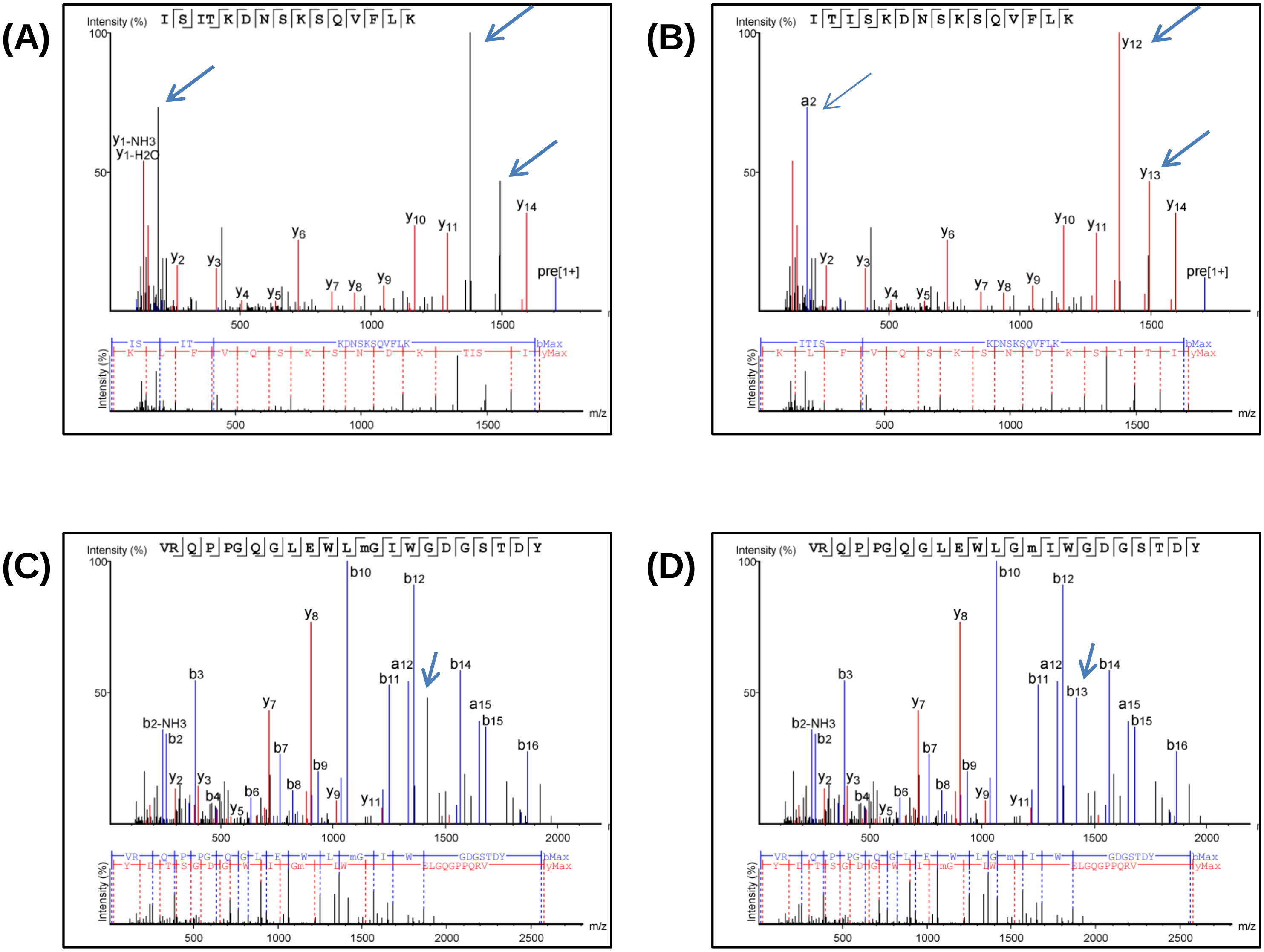

Supplementary Figure S3. High-confidence spectra to support our corrections of the WIgG1 heavy chain. (A) Assignment of residues “ISIT” at positions 67-70 in the WIgG1 heavy chain does not match three peaks in the spectrum. (B) The spectrum is better interpreted with the assignment “ITIS” as in our assembly result. (C) Assignment of residues “MG” at positions 49-50 in the WIgG1 heavy chain does not match one peak in the spectrum. (B) The spectrum is better interpreted with the assignment “GM” as in our assembly result.

(A)

(B)

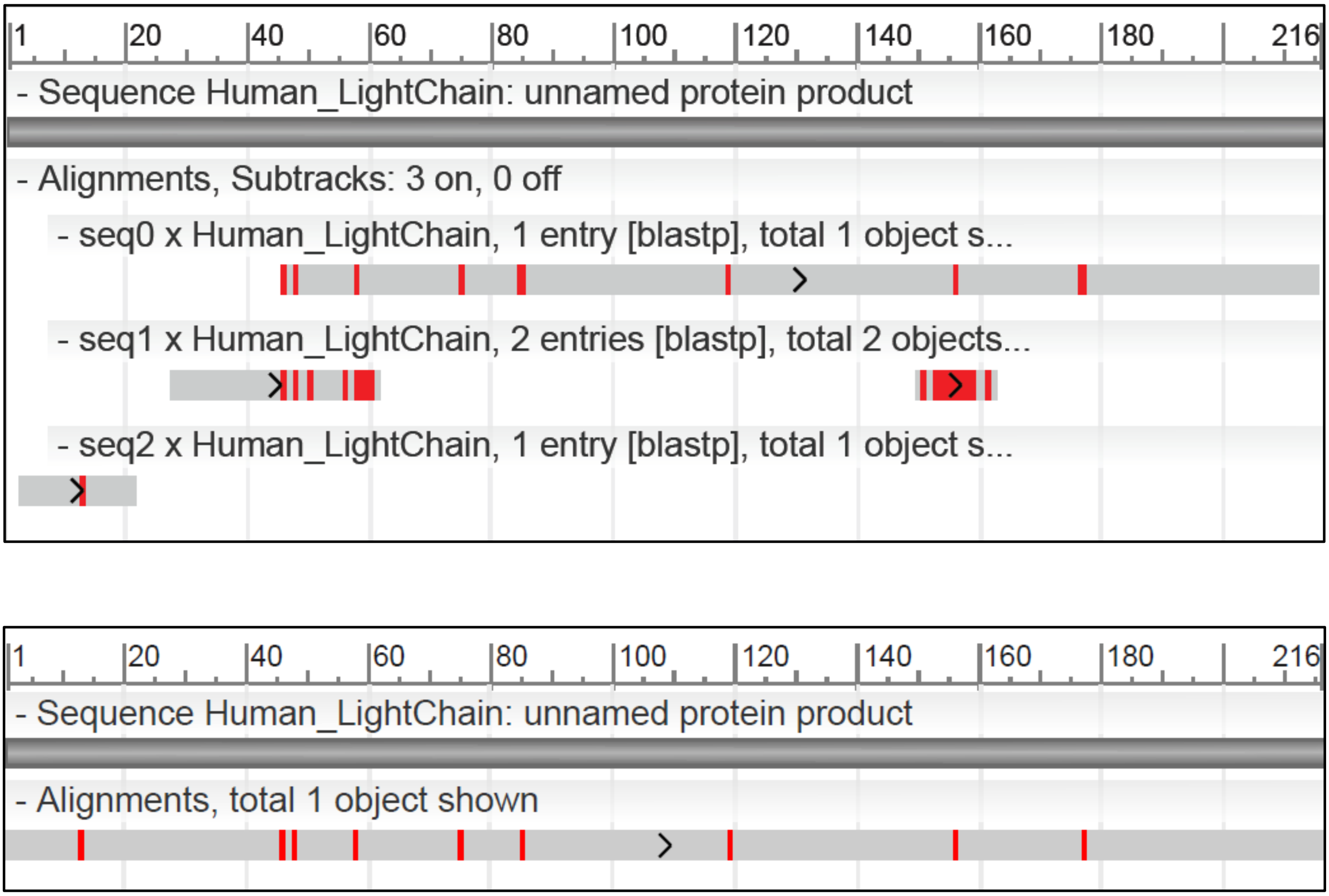

(C)

```
Sbjct  1    ELVLTQSPASLSISPGERATLSCRASQSVSSYLAWYQHKPGQAPRILIYDASTRATGIPA  60
Query  1    ELVLTQSPASLSLSPGERATLSCRASQSVSSYLAWYQHKPGQAPRLLLYDASTRATGLPA  60

Sbjct  61   RFSGSGSGTDFTLTISSLEPEDFAIYYCQQRSNWPPSFTFGPGTRVDLKRTVAAPSVFIF  120
Query  61   RFSGSGSGTDFTLTLSSLEPEDFALYYCQQRSNWPPSFTFGPGTRVDLKRTVAAPSVFLF  120

Sbjct  121  PPSDEQLKSGTASVVCLLNNFYPREAKVQWKVDNAIQSGNSQESVTEQDSKDSTYSISST  180
Query  121  PPSDEQLKSGTASVVCLLNNFYPREAKVQWKVDNALQSGNSQESVTEQDSKDSTYSLSST  180

Sbjct  181  LTLSKADYEKHKVYACEVTHQGLSSPVTKSFNRGEC  216
Query  181  LTLSKADYEKHKVYACEVTHQGLSSPVTKSFNRGEC  216
```

Supplementary Figure S4. Assembly results for the HUMAN light chain. (A) BLAST alignment of the top assembled contigs from list PSM-DN against the target light chain. (B) BLAST alignment of the full-length contig assembled from list PSM-DD against the target light chain. (C) Details of the alignment in (B).

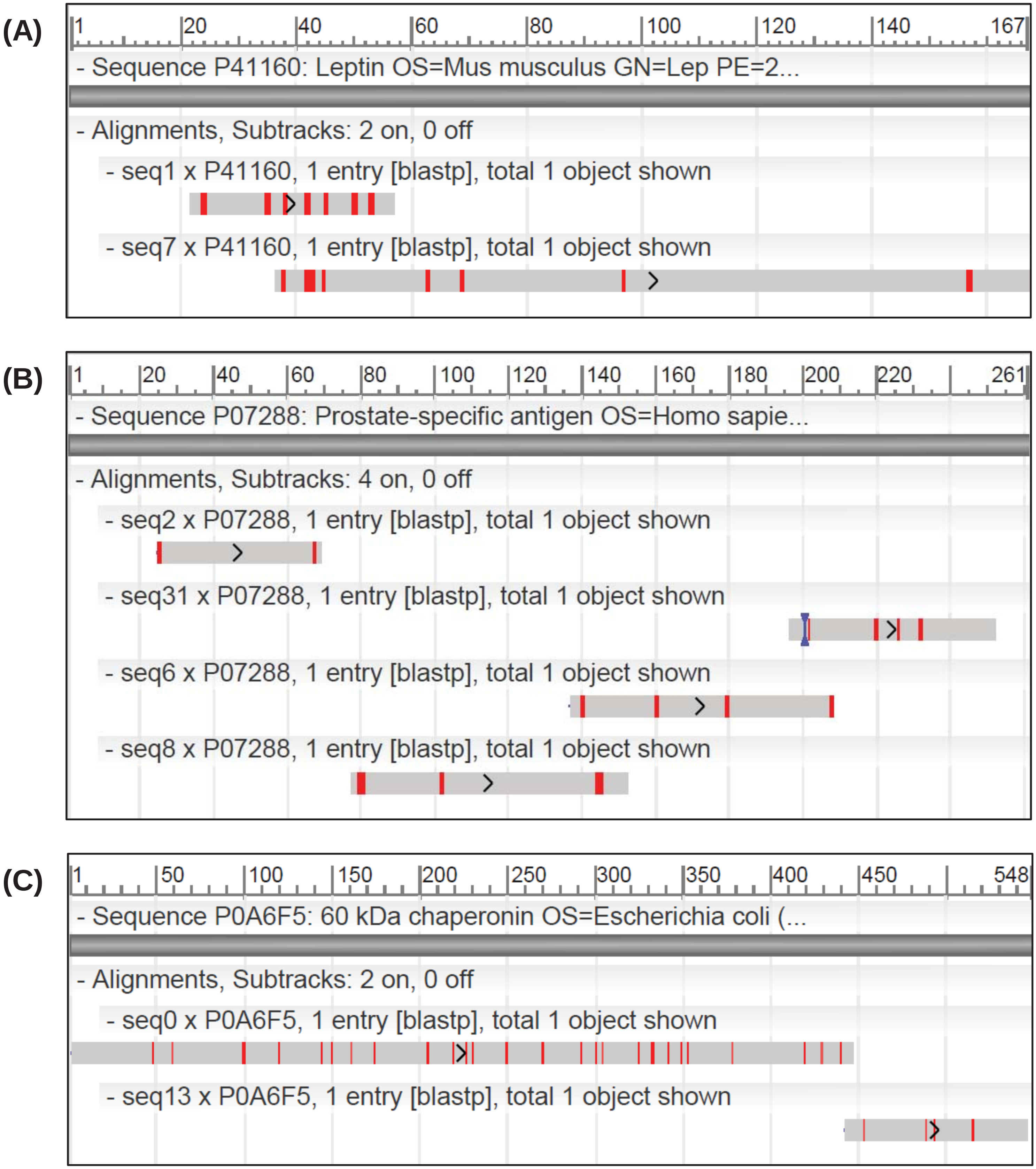

Supplementary Figure S5. BLAST alignments of assembled contigs for the dataset of 6-protein mixture against the respective target protein sequences. (A) leptin. (B) kallikrein. (C) groEL.

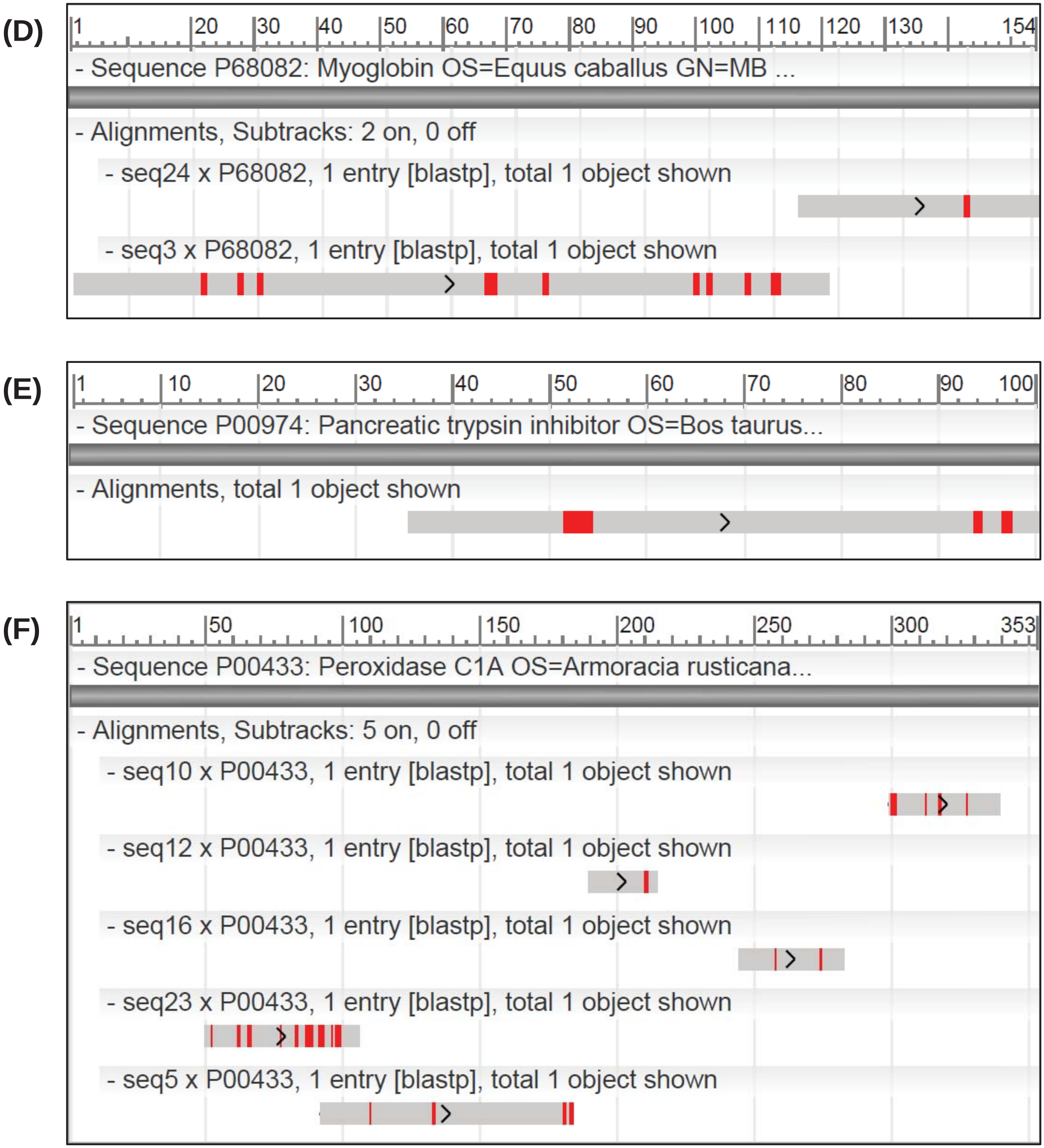

Supplementary Figure S5 (continued). BLAST alignments of assembled contigs for the dataset of 6-protein mixture against the respective target protein sequences. (D) myoglobin. (E) aprotinin. (F) peroxidase.
